# A novel c-di-GMP binding domain in glycosyltransferase BgsA is responsible for the synthesis of a mixed-linkage β-glucan

**DOI:** 10.1038/s41598-017-09290-2

**Published:** 2017-08-21

**Authors:** Daniel Pérez-Mendoza, Daniela Bertinetti, Robin Lorenz, María-Trinidad Gallegos, Friedrich W. Herberg, Juan Sanjuán

**Affiliations:** 1 Department of Biochemistry, University of Kassel, Kassel, Germany; 2 Dpto. Microbiología del Suelo y Sistemas Simbióticos. Estación Experimental del Zaidín, Consejo Superior de Investigaciones Científicas (CSIC), Granada, Spain; 3 Present Address: Institute for Genetics and Cologne Excellence Cluster on Cellular Stress Responses in Aging-Associated Diseases (CECAD), University of Cologne, Cologne, Germany

## Abstract

BgsA is the glycosyltransferase (GT) involved in the synthesis of a linear mixed-linkage β-glucan (MLG), a recently described exopolysaccharide activated by c-di-GMP in *Sinorhizobium meliloti* and other Rhizobiales. Although BgsA displays sequence and structural homology with bacterial cellulose synthases (CS), it does not contain any predictable c-di-GMP binding domain. In this work we demonstrate that the cytoplasmic C-terminal domain of BgsA (C-BgsA) binds c-di-GMP with both high affinity (K_D_ = 0.23 μM) and specificity. C-BgsA is structurally different to the otherwise equivalent cytoplasmic C-terminal domain of CS, and does not contain PilZ motifs for c-di-GMP recognition. A combination of random and site-directed mutagenesis with surface plasmon resonance (SPR) allowed identification of the C-BgsA residues which are important not only for c-di-GMP binding, but also for BgsA GT activity. The results suggest that the C-BgsA domain is important for both, c-di-GMP binding and GT activity of BgsA. In contrast to bacterial CS where c-di-GMP has been proposed as a derepressor of GT activity, we hypothesize that the C-terminal domain of BgsA plays an active role in BgsA GT activity upon binding c-di-GMP.

## Introduction

The second messenger bis-(3′,5′)-cyclic diguanosine monophosphate (cyclic diguanylate, c-di-GMP, cdG) is a highly versatile signalling molecule that controls essential bacterial processes^1, 2^. It is synthesized by diguanylate cyclases (DGC, with GGDEF domains) and degraded by phosphodiesterases (PDEs, with EAL or HD-GYP domains), and sensed by a great variety of c-di-GMP-binding effectors that control diverse targets and functions.

Although several c-di-GMP binding motifs have been described, this second messenger can additionally bind to a diverse range of protein folds which are difficult to predict bioinformatically^3^. The impact of c-di-GMP over a given cellular process is further complicated by the fact that this second messenger can bind multiple receptors within the same biological process, a phenomenon that has been termed ‘sustained sensing’^4^. Furthermore, c-di-GMP exhibits a high structural diversity and flexibility, and can exist in the form of monomers, dimers or even tetramers and with conformations from a fully stacked to an extended form^3^. This strengthens the idea of the existence of complex and diverse c-di-GMP recognition mechanisms in bacteria, thus suggesting that many c-di-GMP binding proteins still remain to be discovered.

So far c-di-GMP effectors include different structural components, transcriptional regulators, transporters, enzymes, and even mRNA riboswitches (recently reviewed in refs 3, 5). From a purely mechanistic perspective, effector proteins could be classified as RXXD-like, EAL domain related, and PilZ domains, plus a broad miscellaneous group likely displaying alternative mechanisms of c-di-GMP binding^3^.

Degenerate GGDEF and EAL domain proteins represent important c-di-GMP receptors in bacteria. In the GGDEF group, the c-di-GMP interacts with a conserved RXXD motif located five residues upstream of the active-site GG(D/E)EF^6, 7^. The RXGD motif of the GIL domain of BcsE proteins could also be included in this group. BcsEs are encoded in several cellulose synthase operons and are c-di-GMP-regulated proteins required for maximal production of bacterial cellulose^8^. These so called “RXXD-like” effectors likely evolved from the allosteric inhibition site (I-site) of originally active DGC that have lost their ability to synthesize c-di-GMP, and include some structurally characterized proteins such as PelD^9^. Among the EAL domain related effectors, different sequence variants of the conserved EXLXR motif of EAL domains belonging to enzymatically active PDE, have been reported. This is the case of the QAFLR motif of FimX-like proteins of different *Xanthomonas* species^10, 11^, and the KVLSR of *Pseudomonas fluorescens* LapD^12^. In addition to those motifs, other residues co-operating in c-di-GMP binding have also been reported in these degenerated EAL domains^11, 12^.

The production of exopolysaccharides (EPS) is a common bacterial process known to be regulated by c-di-GMP, with nearly a dozen examples reported (reviewed in refs 13–15). Cyclic-di-GMP can activate the production of more than one EPS by the same strain, and very often this activation involves the binding of the dinucleotide to one or more of the proteins involved in the synthesis and/or secretion of the EPS^13^. Probably the best known example is the activation of cellulose synthases (CSs) by c-di-GMP. Indeed, this second messenger was originally discovered as an allosteric activator of the *Komagataeibacter xylinus* (formerly known as *Gluconacetobacter xylinus*) CS^16^. A specific domain, designated the PilZ (PF07238) and located in the cytoplasmic C-terminal region of BcsA, was later shown to be involved in the CS activation by c-di-GMP^17^. Since then, PilZ has become the paradigm of the c-di-GMP binding domains, and it can appear stand-alone or in association with diverse functional protein domains^18–22^. PilZ includes two separate motifs: an RXXXR with conserved arginine residues surrounding one guanine base of c-di-GMP, and a (D/N)X(S/A)XXG motif that surrounds the other guanine of the molecule. Indeed, the binding of the second messenger functions as a link between these two motifs, generating a conformational change that initiates downstream signaling events^3^. The molecular mechanism of the CS activation by c-di-GMP through its PilZ domain has been recently revealed in *Rhodobacter sphaeroides* (Rsp^23^). Binding of c-di-GMP to the Rsp BcsA releases an autoinhibited state of the CS, by disrupting a conserved regulatory salt bridge that controls access of the substrate to the active site via a so-called gating loop. Thus, in this protein, PilZ behaves as a repressor domain in the absence of c-di-GMP. In fact, specific mutations interfering with the formation of this regulatory salt bridge result in constitutively active CS variants^23^.

Transcriptional regulators binding c-di-GMP usually contain a helix-turn-helix (HTH) DNA-binding domain but do not present predictable c-di-GMP binding motifs in their sequences^24, 25^. The best studied is FleQ of *Pseudomonas aeruginosa*, where the second messenger interacts with the AAA + ATPase domain, at a site distinct from the ATP binding pocket, but none-the-less disturbing its ATPase activity and therefore its transcriptional role^26^. Interestingly, several recent studies have reported a new set of c-di-GMP effectors associated with other bacterial ATPases^27–30^. These c-di-GMP-binding ATPases are proteins associated with different bacterial secretion systems, flagella and type IV pilus formation, and were shown to specifically bind c-di-GMP with high affinity. However, residues in the described c-di-GMP binding motifs are not well conserved, and even the effect on the ATPase activity can be positive or negative, depending upon the system considered^27, 30^.

Like many other soil bacteria, rhizobia produce a complex array of EPS which provide not only protection against environmental biotic and abiotic stresses, but also play important roles in cell aggregation and biofilm formation; all critical processes during the interaction of bacteria with their legume hosts^31, 32^. The production of some of these EPS, such as cellulose and curdlan, is regulated by c-di-GMP^13^. The model strain *Sinorhizobium meliloti* 8530 (Sme), a nitrogen-fixing symbiont of alfalfa (*Medicago sativa*), lacks the gene clusters for cellulose and curdlan biosynthesis, but produces other EPSs. Recently, an AraC-like transcriptional regulator named CuxR has been reported in Sme to be involved in the regulation of a so far structurally uncharacterized EPS in a c-di-GMP dependent manner^33^. In addition, Sme produces another c-di-GMP-regulated EPS, a linear Mixed-Linkage β-Glucan or MLG, regulated by c-di-GMP at posttranslational level^34^. This unique bacterial EPS resembles other β-glucans found in cereals and certain lichens but has a distinctive primary structure, with a perfect β (1 → 3)/β (1 → 4) glycosidic bond alternation between the glucose residues^34^. MLG participates in aggregation and biofilm formation and is required for efficient attachment of Sme to the roots of its host plant, resembling the biological role of cellulose in other bacteria^34^. The two MLG biosynthetic genes so far identified seem widespread amongst rhizobial and non-rhizobial species^34^. BgsA is the glycosyltransferase (GT) involved in MLG biosynthesis and displays sequence and structural homology with CSs. BgsA contains seven predicted transmembrane helices (TMH) with a central CESA_CelA_like domain (cd06421) between TMH3 and TMH4, likely involved in the elongation of the glucan chain using UDP-glucose as a substrate^34^. BcsA and BgsA proteins both contain a C-terminal cytoplasmic tail, much larger in BcsAs than in BgsAs. However, only the one found in CS contains a PilZ domain involved in binding to c-di-GMP. Although BgsA does not seem to contain any predictable c-di-GMP binding domain, its interaction with c-di-GMP has been proposed^34^.

In this work, we demonstrate that the C-terminal cytoplasmic tail of BgsA binds c-di-GMP with both high affinity and specificity. A combination of random and site-directed mutagenesis with surface plasmon resonance (SPR) has allowed the identification of amino acids in this C-terminal tail which are important for BgsA activity, for binding to c-di-GMP, and for both. The results suggest that C-BgsA has an important role in GT activity, in addition to binding to c-di-GMP.

## Results

### The C-terminal domain of BgsA binds specifically to c-di-GMP

**Figure 1:**
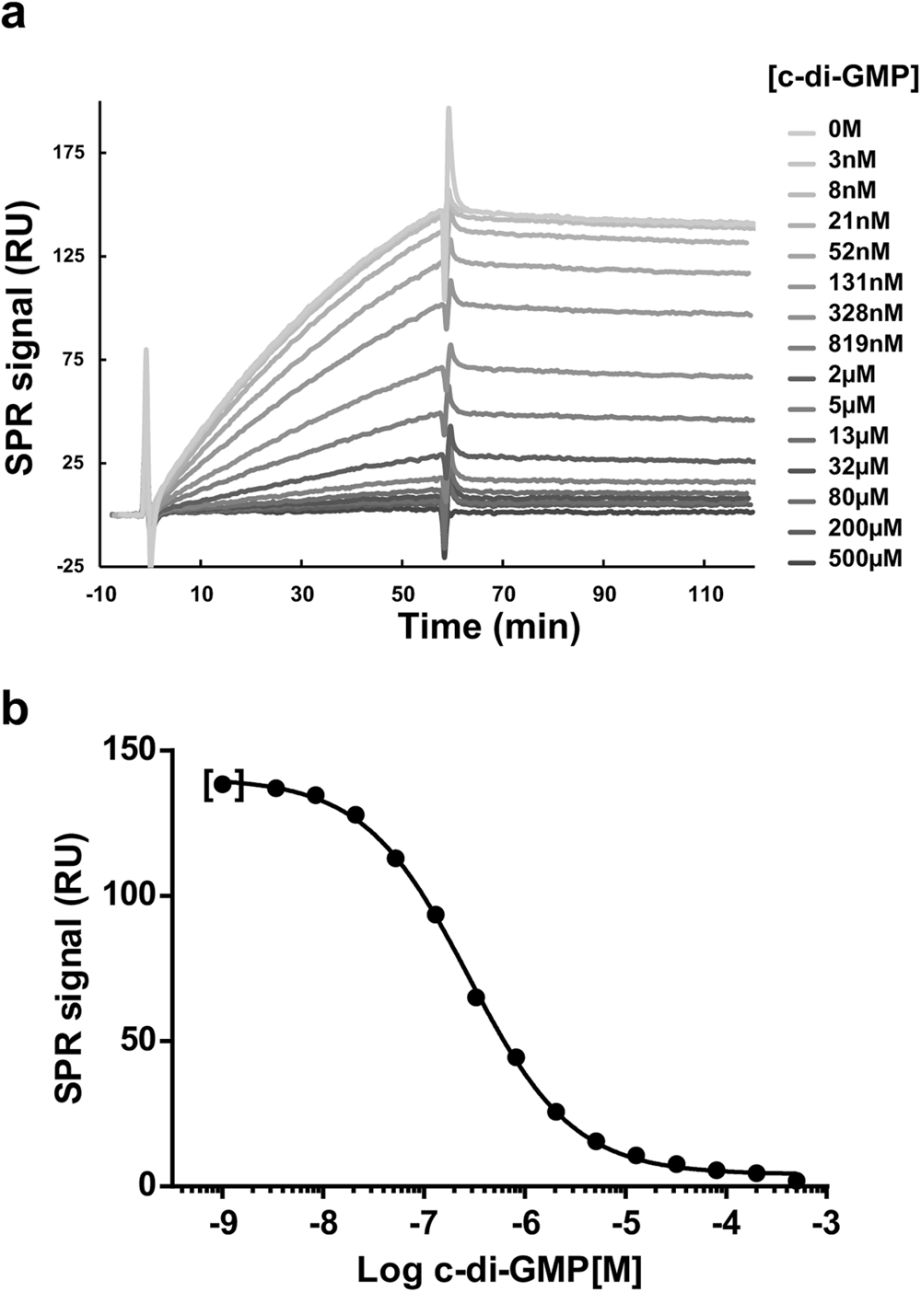
Competition experiments with increasing concentrations of free c-di-GMP in SPR binding assays of C-BgsA to 2′-AHC-c-di-GMP sensor chip. (**a**) Representative SPR sensograms after buffer subtraction of C-BgsA with the different c-di-GMP concentrations. 25 nM of C-BgsA was incubated with increasing concentrations of free c-di-GMP (from 0 to 500 µM) for 2.5 h and then injected over a high density 2′-AHC-c-di-GMP sensor chip surface. Time point was set up to zero at the injection point. A report point was set directly before the end of the association phase to monitor the SPR signal of each sample. (**b**) The SPR report point represented in (**a**) was plotted against the logarithmic c-di-GMP concentration and the data was fitted with a sigmoidal dose-response curve. First point indicated within brackets corresponds to 0 M of c-di-GMP.

Earlier experiments with 2′-Fluo-AHC-c-di-GMP suggested that the C-terminal cytoplasmic tail of BgsA could be involved in c-di-GMP recognition^34^. To further elucidate this, the 139 aa C-terminal tail of BgsA (C-BgsA) was expressed and purified by affinity chromatography, and tested for c-di-GMP binding using surface plasmon resonance (SPR) assays. In these experiments, C-BgsA bound to the 2′-AHC-c-di-GMP sensor chip in a concentration-dependent manner with an equilibrium dissociation constant (K_D_) of 0.23 μM. Binding affinity to free c-di-GMP was also evaluated in solution competition experiments, combining 25 nM C-BgsA with increasing concentrations of c-di-GMP (from 0 to 500 µM). Here, C-BgsA showed a similar affinity for free c-di-GMP, with an EC_50_ of 0.27 ± 0.01 μM (Fig. 1). In a parallel control competition experiment, binding of C-BgsA to the sensor chip surface remained unaffected when 500 µM cGMP was added, which indicates that C-BgsA does not bind this mononucleotide. When C-BgsA was injected with 500 µM of c-di-AMP, a certain degree of competition was observed with a reduction of 17% in C-BgsA binding to the c-di-GMP chip. Nevertheless, the affinity for c-di-AMP is very low compared to that for c-di-GMP, as using the same concentration of c-di-GMP (500 µM) resulted in a 98% reduction of the binding signal (Fig. 1). These experiments clearly demonstrate that C-BgsA selectively binds c-di-GMP with a sub-micromolar affinity.

### BgsA does not contain a typical PilZ domain for binding c-di-GMP

**Figure 2:**
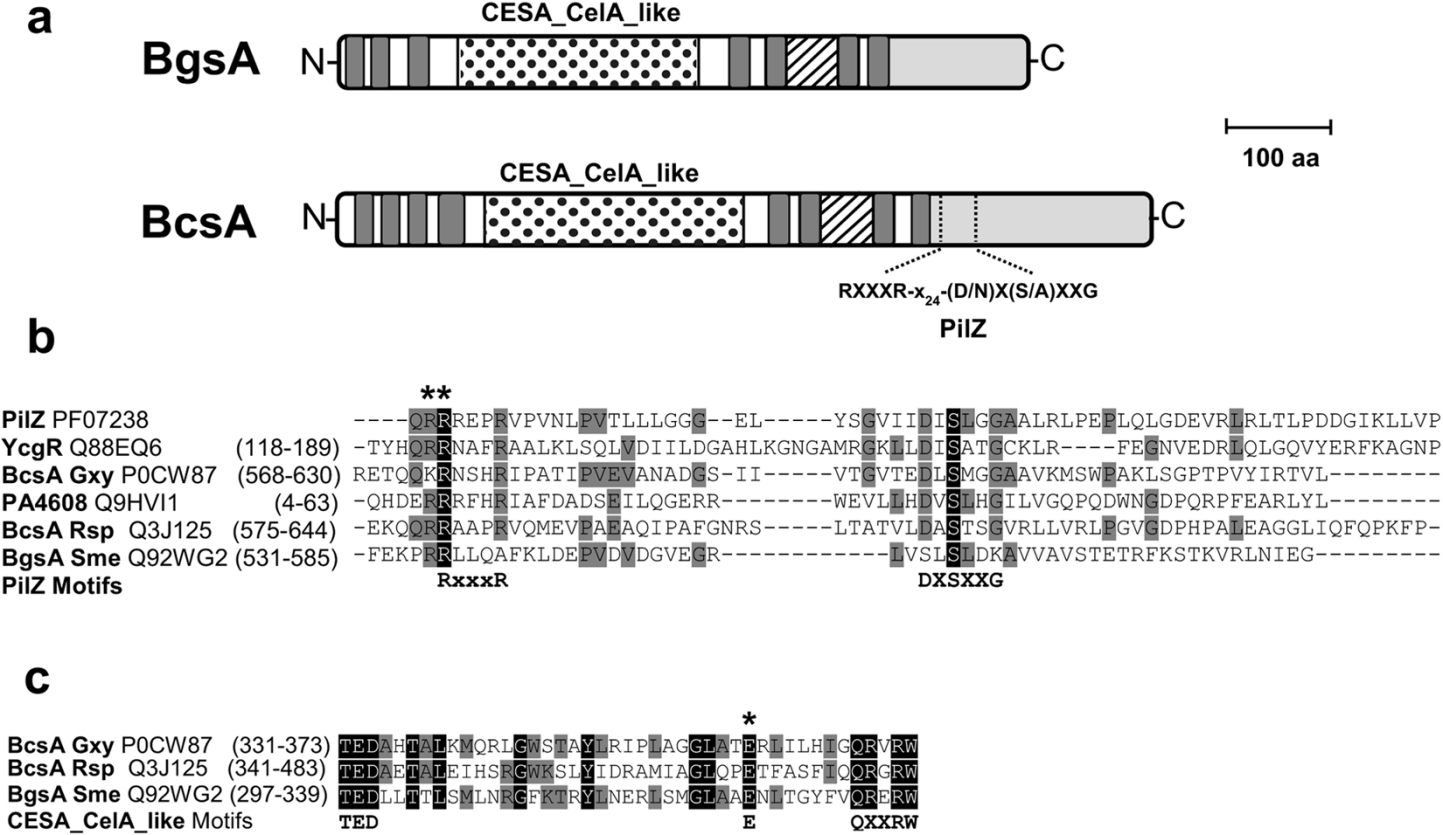
Similarities between BgsA and bacterial cellulose synthases. (**a**) Scale schematic diagram of homology between cellulose synthase of *Rhodobacter sphaeroides* BcsA, and BgsA of *Sinorhizobium meliloti*. Transmembrane helices (TMHs) in BcsA and predicted TMHs in BgsA (TMHMM; ref. 49) are depicted as dark grey bars. The predicted C-terminal cytoplasmic segment of each protein is represented in light grey. The putative gating loop containing FxVTxK motif and CESA_celA_like catalytic domain of each sequence are indicated by striped and dotted segments, respectively. The two principal PilZ motifs implicated in the c-di-GMP recognition (RXXXR and (D/N)X(S/A)XXG) are indicated in BcsA. (**b**) Alignment of C-BgsA with PilZ domains of different proteins. The two BgsA arginines (R535& R536) potentially corresponding to R580 of Rsp BcsA involved in the salt bridge formation and binding to c-di-GMP^23^ are indicated with an asterisk. (**c**) Alignment of a conserved region of the CESA_celA_like catalytic domain of BgsA and two well characterized cellulose synthases. The glutamic acid implicated in the salt bridge formation for the auto inhibition of BcsA in the absence of c-di-GMP, is indicated with an asterisk. Other well characterized motifs (TED and QXXRW) of the catalytic domain of glycosyltransferases are also indicated. Color codes: white on black background = invariant residues; black on dark grey = strongly conserved and black on white = non-conserved.

BgsA displays similarities with other c-di-GMP-activated GTs. Besides the membrane spanning domains, it also contains a central CESA_CelA_like domain (cd06421) present in GTs using UDP-glucose as a substrate and involved in the elongation of the glucan chain of unbranched polymers such as cellulose (Fig. 2a). This BgsA domain shares significant sequence similarity with bacterial CSs: e.g. 44% and 40% of identity with CESA_CelA_like domains of the well characterized CSs of *K. xylinum* (aa 150 to 381) and *R. sphaeroides* (aa 140 to 391). BgsA likely also contains the so-called gating loop between TMH5 and TMH6, including its representative motif FXVTXK (Fig. 2a; ref. 23). However, sequence conservation between BgsA and CSs drastically drops after the final transmembrane domain and BLAST tools^35^ fail to recognize any significant sequence similarity between the cytoplasmic C-terminal domain of BgsA and bacterial CSs. Besides the C-terminal tail of CS being significantly larger, the C-terminus of BgsA does not seem to contain any recognizable PilZ motifs, which in bacterial CSs are implicated in binding to, and subsequent de-repression by, c-di-GMP. Prediction of the secondary structure of C-BgsA is also different from the typical six-stranded β-barrel structure described for the PilZs of CSs (Supplementary Fig. S1; refs 23, 36). Indeed, most of the key PilZ residues [motifs RXXXR and (D/N)X(S/A)XXG] important for binding to c-di-GMP^17^ are missing in BgsA (Fig. 2b). Nonetheless, BgsA might contain a few of the conserved residues involved in the c-di-GMP activation mechanism described for BcsA of Rsp^23^. In this well characterized CS, c-di-GMP releases an autoinhibited state of the enzyme by breaking a conserved regulatory salt bridge that controls access of the substrate to the active site via a so-called gating loop^23^. This salt bridge is established between a glutamate residue located in the CESA_CelA_like domain (E371) and the first arginine residue (R580) of the RXXXR motif of the PilZ domain of BcsA. The change of R580 to alanine (R580A) renders BcsA constitutively active in the absence c-di-GMP^23^. We observed that BgsA contains a glutamate (E327) which could correspond to Rsp BcsA E371 (Fig. 2c) and two arginines (R535A & R536A), potentially corresponding to Rsp BcsA R580 (Fig. 2b). In order to test if BgsA exhibits a similar repression mechanism in the absence of c-di-GMP, we obtained mutants in either one (R536A) or both (R535A & R536A) of the consecutive BgsA arginine residues (Fig. 2b). As in the study of Morgan *et al*.^23^, we replaced these Arg for Ala, and determined the impact of these changes in the c-di-GMP dependent production of MLG. However, neither of these mutations appeared to have a significant effect on MLG synthesis by BgsA under high c-di-GMP levels (Sme *bgsA* Tn7::pleD*Tc, Table S1), as glucan production levels were similar to the wild type BgsA (Supplementary Fig. S2). Under physiological levels of c-di-GMP no significant production of MLG was observed with either C-BgsA mutant, similar to the wild type (Supplementary Fig. S2). These results further supported the notion that BgsA does not contain a PilZ-like c-di-GMP binding domain.

### Random mutagenesis of C-BgsA by error-prone PCR for the identification of mutants affected in MLG production

In order to gain insights about the residues of C-BgsA important for the recognition of c-di-GMP and subsequent activation of MLG production, a random mutagenesis approach was set up. Two primers flanking the coding region of C-BgsA were used in an error-prone PCR strategy optimized to introduce a tractable number of random mutations in this domain. Based on a plasmid harboring the complete sequence of *bgsA* (pBBR5BgsA), a mutant library was created by exchanging the AsiSI/EcoRI fragment encoding the wild type C-BgsA domain with the randomly mutagenized PCR products. This C-BgsA mutant library was introduced *en masse* into a *bgsA* null mutant background expressing high c-di-GMP levels (Sme *bgsA* Tn7pleD*Tc). Following a functional screening for MLG production using congo red plates (CR), three different types of colonies could be visualized: (i) the great majority were red colonies (CR^+^), similar to the one expressing pBBR5BgsA (wild type); (ii) white colonies likely expressing a non-functional BgsA, similar to the negative control (i.e., the empty vector pBBRMCS-5); and (iii) pink colonies, suggestive of transconjugants expressing partially active BgsA variants.

The plasmids from 29 white or pale pink colonies were extracted and the fragments encoding C-BgsA domains were sequenced to identify mutations. In 4 white clones (H14B, G12B, G3B and H26B) the mutations generated early stop codons, leading to truncated versions of BgsA lacking the final 41, 56, 102 or 150 amino acids, respectively (Supplementary Fig. S3). These non-functional, C-terminally truncated versions of BgsA indicated that this cytoplasmic segment is essential for GT activity. Also, all truncated C-BgsA variants did not show any appreciable binding to 2′-Fluo-AHC-c-di-GMP in a dot-blot assay (Supplementary Fig. S3), further supporting the requirement of c-di-GMP binding for significant BgsA activity.

The 25 remaining plasmids encoded full-length C-BgsA peptides but their sequences revealed the presence of a variable number of nucleotide substitutions, ranging from 1 to 9 per clone, which hindered functional assignment of the residues involved. Only two clones harboured a single mutation and in both cases, the changes had affected the same position, glycine 635 [clones G20I (G635R) and G25I (G635V)]. These two BgsA mutants produced pale pink colonies on CR plates, suggesting some residual capacity for MLG production. As expected, complementation of a *bgsA* mutant (Sme *bgsA* Tn7::pleD*Tc) with the G20I and G25I plasmids produced a CF derived fluorescence that was just 16.8 ± 0.75% (G20I) and 14.9 ± 0.68% (G25I) of that shown by the strain complemented with the wild type BgsA. These results indicated that substitution of the glycine 635 by an arginine or a valine residue strongly diminished but did not abolish the GT activity. To determine whether this reduction of activity correlated with a lower c-di-GMP-binding capacity, the G20I and G25I C-BgsA domains were subcloned into the pQE-80L expression vector, overexpressed and purified in *E. coli*. Affinities of the G20I and G25I mutants for the c-di-GMP chip were then compared to the wild type. The K_D_ obtained for G20I (0.19 μM) and G25I (0.14 μM) was similar to the wild type (0.23 µM), indicating that the reduction in BgsA GT activity and MLG production was not caused by a defect in c-di-GMP binding.

**Figure 3:**
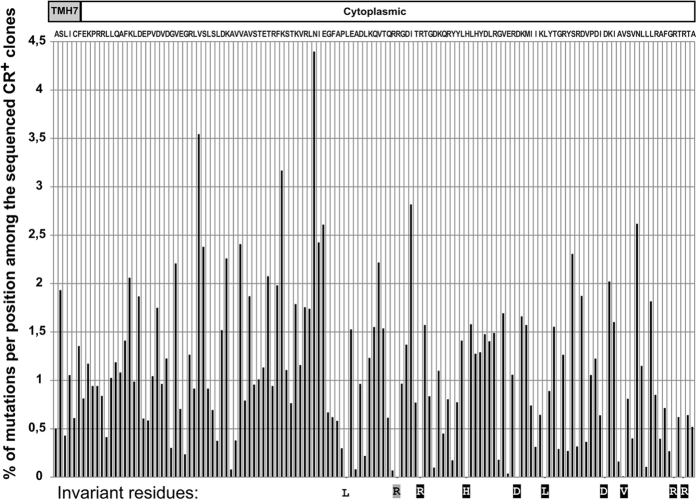
Random mutagenesis of the C-BgsA domain. After error-prone PCR mutagenesis, the C-BgsA domains of one thousand clones retaining MLG production (CR^+^, see text) were Illumina deep sequenced. In the figure, the proclivity of every position in C-BgsA to be mutated without affecting MLG production is indicated as the rate (%) of mutations. Last predicted transmembrane helix (TMH7) is shown. The BgsA sequence is indicated on the top, and the invariant residues (0% of changes) in the mutagenized CR^+^ population are shown at the bottom of the graph. Color background of these invariant residues indicate their conservation in BgsA orthologous sequences available in data bases (black, fully conserved; grey, highly conserved; white, not conserved; see also Supplementary Fig. S4).

After the limited success of the above approach to identify residues in the C-BgsA domain important for BgsA GT activity and c-di-GMP binding, and being aware that most of the mutant clones produced wild type-like CR^+^ red colonies, we reasoned that these CR^+^ clones could provide valuable information about the C-terminal tail residues dispensable for BgsA activity. Mutant but yet CR^+^ clones would also indirectly inform about those C-BgsA residues essential (for which no variation is allowed) for BgsA to attain high activity. First, plasmids from 26 individual CR^+^ colonies were isolated and sequenced. We found a significant average of 3 point mutations per clone, ranging from none to 6. Then, one thousand functional CR^+^ colonies (Sme *bgsA* Tn7pleD*Tc pBBR5BgsA*) arisen from the above PCR mutagenesis, were pooled, grown and plasmid DNA isolated. In a parallel control, the strain containing the non-mutated wild type BgsA plasmid (Sme *bgsA* Tn7pleD*Tc pBBR5BgsA) was prepared similarly. Plasmid DNA from both cultures was paired-end sequenced by Illumina, using 2 primers flanking the coding sequence of the C-BgsA domain. After deep sequencing analysis, the 2,183 full-length sequences obtained were translated and the frequency of change for each residue in the C-BgsA domain was calculated. To minimize the effects of sequence variations artifactually raised by the intrinsic errors of the sequencing technique, the values obtained in the non-mutagenized control population (pBBR5BgsA) were subtracted from the values obtained for the corresponding positions in the mutant population (pBBR5BgsA*). Among the mutagenized yet CR^+^ clones, we found that the net variation rate per any given aa position ranged from 0 to 4.5%. It should be considered that certain mutations can lead to conservative changes which do not affect BgsA activity, and some mutations detrimental for activity may be compensated by additional mutations in certain BgsA-active clones. Nevertheless, residues essential for high BgsA activity must be less prone to substitution among those BgsA mutants retaining full or nearly full MLG synthesis. We found that only 10 of the 139 C-BgsA residues remained unchanged (0% variation) in all the 1,000 mutant yet CR^+^ clones sequenced (Fig. 3). Interestingly, 8 out of those 10 positions also corresponded to residues invariant amongst naturally-occurring C-BgsA sequences encoded in diverse bacterial genomes available in databases (Supplementary Fig. S4), further suggesting their importance for BgsA activity. Moreover, these highly conserved residues are not evenly distributed along the C-BgsA domain, but are localized towards the C-terminus of the C-BgsA tail (Fig. 3 and Supplementary Fig. S4).

### Identification of C-BgsA residues involved in c-di-GMP binding and BgsA activity

**Figure 4:**
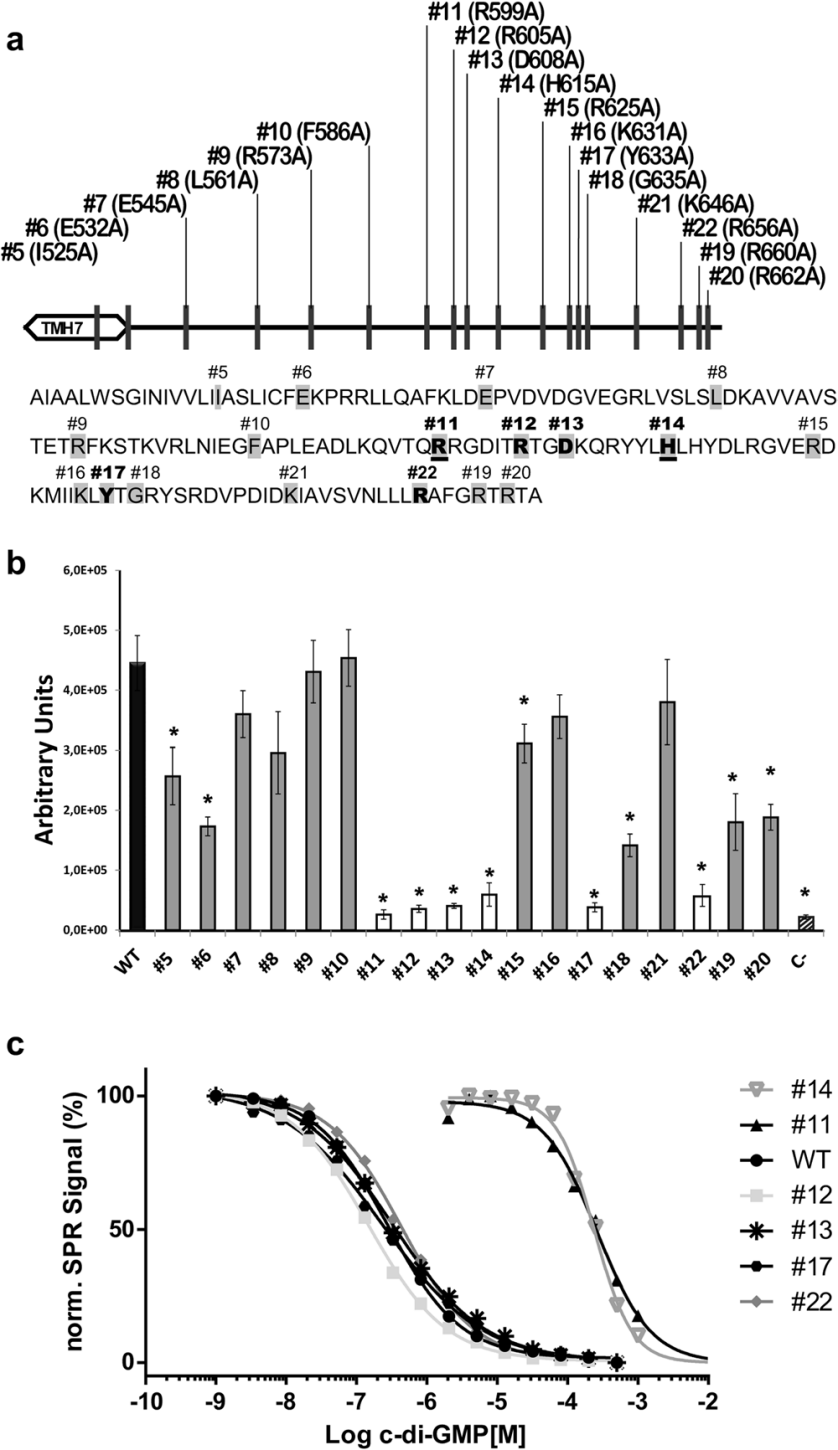
Effect of site-directed mutants on C-BgsA binding to c-di-GMP and BgsA activity. (**a**) Last predicted transmembrane helix of BgsA is indicated (TMH7). Positions mutated to alanine are in grey background. Those that showed MLG production <10% of the wild type (**b**) are bold faced and those affected in the c-di-GMP binding (**c**) are underlined. (**b**) Quantification of MLG production as CF-derived fluorescence in *S. meliloti* 8530 *bgsA* under high intracellular c-di-GMP conditions (Tn7::Tc pleD*) complemented with each of the 18 C-BgsA mutants indicated in (**a**). White bars depict mutants with less than 10% of the wild type MLG production. The wild type BgsA and the negative control (C-, empty vector pBBRMCS-5) are indicated with solid and striped black bars, respectively. Results are expressed in arbitrary units ± SD from four independent biological replicates. Asterisks indicate significant differences with the wild type on statistical homoscedastic two-tailed student’s t-test with P < 0.01. (**c**) Representative affinity fit for SPR competition experiments performed with 25 nM of C-BgsA wild type and mutants #12, #13, #17, #22 incubated for 2.5 hours with increasing c-di-GMP concentrations, from 0 to 500 µM, and 100 nM of mutants #11 and #14 incubated for 2.5 hours with increasing c-di-GMP concentrations, from 0 to 1 mM. Sigmoidal dose-response curves for #11 and #14 were extrapolated and 0 RU were set to 0%. The different c-di-GMP concentrations are represented in Log scale and the SPR signal of the wild type and different mutants are normalized in %.

The information acquired from the above experiments (Fig. 3 and Supplementary Fig. S4) was used to select C-BgsA residues for site-directed mutagenesis. Particular attention was also paid to arginine residues since this amino acid is frequently involved in c-di-GMP binding^3^. Eighteen individual point mutations to alanine were generated along the C-terminal domain of BgsA (Fig. 4a). The 18 mutant plasmids were introduced by conjugation into the Sme *bgsA* Tn7pleD*Tc strain and MLG production was quantified by the CF-derived fluorescence in liquid media (Fig. 4b). As shown in Fig. 4, the MLG production by 6 of the 18 mutants (#7, 8, 9, 10, 16 and 21) was not significantly affected (P < 0.01), whereas 6 others produced around 50% of the wild type (#5, 6, 15, 18, 19 and 20). Interestingly, the remaining 6 mutants seemed profoundly affected, with MLG production ranging from none to less than 10% of the wild type: #11, #12, #13, #14, #17 and #22 (Fig. 4b). The C-BgsA domains of these profoundly affected mutants were subcloned into the pQE-80L expression vector and the respective proteins overexpressed and purified by affinity chromatography (Supplementary Fig. S5). A dot-blot assay with 1.5 µg of each purified protein showed no detectable binding to the 2′-Fluo-AHC-c-di-GMP of mutants #11 and #14. The rest of the mutants (#12, #13, #17 and #22) were undistinguishable from the wild type (Supplementary Fig. S5). This observation was further confirmed by SPR competition experiments with free c-di-GMP. The affinity of the mutants #12, #13, #17 and #22 for c-di-GMP was similar to that of the wild type C-BgsA, with EC_50_ values of 0.14 ± 0.01 μM, 0.27 ± 0.07 μM, 0.23 ± 0.01 μM and 0.40 ± 0.05 μM, respectively. However, affinity for c-di-GMP of mutants #11 and #14 was reduced by about 1000-fold, with EC_50_ values of 372 ± 114 µM and 292 ± 73 µM, respectively (Fig. 4c). These mutants were affected at two relatively close residues, R599 and H615. In mutant #11 the change involved an arginine (R599A), an amino acid frequently involved in c-di-GMP binding in several types of effectors^3^. R599 is located in a predicted loop without a regular secondary structure (Supplementary Fig. S1). However, in #14 the mutation involves a histidine (H615A) located in the center of a predicted β-strand and renders a similar effect in c-di-GMP binding and BgsA activity as #11. Additionally, we identified several mutants (R605A, D608A, Y633A, R656A) which abrogated more than 90% BgsA activity (Fig. 4b) without strongly affecting the binding to c-di-GMP (Fig. 4c), suggesting that c-di-GMP binding to C-BgsA is necessary but not sufficient for BgsA GT activity and that C-BgsA may play an active role in BgsA GT activity upon binding c-di-GMP.

## Discussion

The bacterial second messenger cyclic diguanylate regulates biosynthesis of diverse EPS, often by binding of c-di-GMP to one or more proteins in the EPS biosynthetic complex^13^. For the recently discovered MLG, posttranslational regulation through allosteric activation of the GT BgsA was suggested^34^, which was intriguing since BgsA does not contain any previously described c-di-GMP binding domain^34^.

In the present work, biomolecular interaction studies have clearly demonstrated that the cytoplasmic C-terminal segment of BgsA specifically recognizes c-di-GMP with submicromolar affinity, which is comparable to other c-di-GMP effectors, i.e. PilZ or the recently discovered MshEN domain^18, 30^, and is also consistent with bacterial intracellular c-di-GMP concentrations which have been estimated to be in the submicromolar to low micromolar range^37, 38^.

The simultaneous loss of both c-di-GMP binding and MLG production in several C-terminal truncated versions of BgsA, as well as in point R599A and H615A mutants, strongly support that the binding of the dinucleotide to C-BgsA is essential for BgsA GT activity. Regarding c-di-GMP binding, only R599 and H615 were found to be involved in c-di-GMP recognition, and substituting either of these two residues for alanine seriously compromised the affinity of the protein for the dinucleotide. This region does not seem to contain any previously described c-di-GMP binding motifs. Arginine 599 in combination with D602 could form a hypothetical RXXD signature, which is one of the simplest c-di-GMP binding motifs frequently located in the allosteric inhibitory site (I-site), five residues upstream of the active-site GG(D/E)EF motif, of different active and inactive DGCs^6, 7^. An RXXD motif is also important for c-di-GMP binding by the GIL domain identified in BcsE proteins^8^. However, D602 showed a relatively high substitution rate (>1%) in the randomized mutant library (Fig. 3) without apparently affecting MLG production. Moreover, this aspartate residue seems conserved only in *Sinorhizobial*/*Ensifer* BgsA sequences, but it is replaced by serine in many other BgsA sequences (Supplementary Fig. S4). This suggests that, in contrast to R599, D602 may not be important for BgsA activity. On the other hand, another nearby RXXD motif (R605-T-G-D608) located within this BgsA region appeared highly conserved in our randomized mutant library as well as in naturally occurring BgsAs (Fig. 3 and Supplementary Fig. S4). Site-directed mutation of those residues (mutants #12 and #13, Fig. 4a) evidenced their importance for MLG production (Fig. 4b) but not for c-di-GMP binding (Fig. 4c). Another amino acid that proved to be important for c-di-GMP recognition was H615 located in a predicted β-strand (Supplementary Fig. S1). The mutant H615A displayed 1,000 times lower affinity for c-di-GMP than the wild type C-BgsA (Fig. 4c). Interestingly, H201 of YcgR (PP4397) from *Pseudomonas putida* has been also reported as an important residue for c-di-GMP recognition; participating in direct H-bonds with one of the two intercalated c-di-GMP molecules^39^. Furthermore, H201 is located on a β-strand and its 3 following residues ‘HLHY’ are also conserved in BgsA (Supplementary Fig. S1; ref. 39), suggesting a possible conserved c-di-GMP recognition motif.

On the other hand, we identified several residues which seem unimportant for c-di-GMP binding but critical for GT activity. This was the case of mutations in G635 (changes to valine, arginine or alanine led to 15–30% of the wild type MLG production), and for the mutants R605A, D608A, Y633A or R656A, which displayed less than 10% of the wild type MLG production while maintaining high affinity for c-di-GMP. The results suggest that C-BgsA may itself have a role in BgsA activity in addition to the essential binding to the cyclic dinucleotide. This is in contrast to what has been reported for the C-terminal segment of BcsA CS, where certain mutations lead to significant GT activity in the absence of c-di-GMP^23^, which suggests that c-di-GMP is a derepressor rather than an activator of CS^40^. It shall be interesting to know the activity and the c-di-GMP dependency of BcsA versions lacking the cytoplasmic C-terminal domain. As reported here, none of C-BgsA mutants showed significant MLG biosynthesis in the absence of c-di-GMP, whereas several C-BgsA mutants that had lost most GT activity still retained wild type affinity for c-di-GMP. This suggests that the mechanism of activation of BgsA by c-di-GMP is different from that reported in CS^23, 40^.

MLG coexists with other c-di-GMP regulated D-glucose homopolysaccharides, such as cellulose or curdlan, whose biosynthetic genes are present in many available Rhizobiales genomes. The c-di-GMP activation mechanism of curdlan synthesis is still unknown^41^. A previously reported phylogenetic study of their respective GTs, suggests that curdlan (CrdS) and MLG synthases (BgsA) share a common ancestor that diverged from CSs (CelA)^34^. The central CESA_CelA_like domain (cd06421) present in all these 3 GTs and involved in the EPS polymerization is highly conserved. In stark contrast, the C-terminal cytoplasmic tail involved in at least 2 of their c-di-GMP regulatory mechanisms (MLG and cellulose synthases), greatly diverged. This may allow differential posttranslational regulatory mechanisms for the production of otherwise coexisting exopolysaccharides in many bacterial species.

A profound study of BgsA tridimensional structure and its interaction with c-di-GMP will be required to determine the stoichiometry, the details of its activation and the actual role of C-BgsA in GT activity.

## Methods

### Bacteria and culture conditions

Bacterial strains and plasmids used in this work are listed in Supplementary Table S1.

Overnight cultures of *Escherichia coli* were routinely grown in Luria–Bertani broth (LB; containing 10 g/L tryptone, 5 g/L yeast extract, 5 g/L NaCl) at 37 °C. Starting cultures of *Sinorhizobium meliloti* 8530 (Sme) strains were grown overnight at 28 °C on TY broth (tryptone-yeast extract-CaCl_2_^42^). MM medium^43^ was used for rhizobial strains in Congo Red (CR) and Calcofluor (CF) assays. When required, antibiotics and other compounds were added at the following final concentrations: Tetracycline (Tc), 10 μg ml^−1^; Ampicillin (Ap) 100 μg ml^−1^; Gentamycin (Gm), 30 μg ml^−1^; CR, 125 μg ml^−1^; CF, 100 µM; Isopropyl β-D-1-thiogalactopyranoside (IPTG) 1 mM.

### Recombinant DNA techniques and sequence alignments

Molecular biology techniques were performed according to standard protocols and manufacturer’s instructions. For complementation experiments, Sme *bgsA* mutant strains with high (Sme *bgsA* Tn7pleD*Tc) and its respective control with physiological levels of c-di-GMP (Sme *bgsA* Tn7Tc) were constructed by using a mini-Tn7 transposon in triparental matings as previously described^44^. The sequences encoding desired mutant versions of C-BgsA domain were subcloned into the expression vector pQE-80L by the PCR amplification using corresponding pBBR5BgsA* plasmid as DNA template, and the primers K91PilZ-F (TAAAGGATCCGCCTCGCTTATCTGCTTC) and K91PilZ-R (TTTTAAGCTTTCCCGCACGCTCTATGC). The PCR DNA fragments were digested with BamHI/HindIII, purified and subcloned into pQE-80L, previously linearized with the same restriction enzymes. Correct constructions were confirmed by sequencing (GATC biotech).

Sequences for BgsA homologues were obtained from NCBI data base after a Blast-P search with BgsA sequence^35^. Alignments of the predicted C-terminal cytoplasmic segments were performed by ClustalW^45^

### Construction of site-directed mutants in C-BgsA

The single mutant BgsA R536A was generated by using the QuickChange® PCR method (Stratagene). Manufacturer’s instructions were followed using 5 ng of pBBR5BgsA DNA as template and primers BgsA R536A-F (GAAGCCGCGGGCCCTGCTGCAGGC) and BgsA R536A-R (GCCTGCAGCAGGGCCCGCGGCTTC). Mutagenesis was confirmed by sequencing (GATC biotech). The same procedure was subsequently used to generate the double mutant R535A & R536A, by using pBBR5BgsA R536A DNA as template and primers BgsA R535A/36A-F (GCTTCGAGAAGCCGGCGGCCCTGCTGCAGGCGTTC) and BgsA R535A/36A-R (GAACGCCTGCAGCAGGGCCGCCGGCTTCTCGAAGC). To avoid any undesired mutation in the backbone, the sequenced AsiI/EcoRI fragments containing the mutations were subcloned into a new pBBR5BgsA backbone. The rest of site-directed mutants were ordered from GenScript (GenScript; Pescataway, NJ, USA).

### Overexpression and purification of C-BgsA constructs

Overnight cultures of OmniMAX *E. coli* strain containing pQE80L::C-BgsA plasmid or the different mutant versions were diluted 1/50 in fresh LB with Ap. Protein expression was induced at an OD_600_ of 0.5–0.6 by adding 1 mM isopropyl β-D-1-thiogalactopyranoside (IPTG). Cells were grown for another 4 hours at 30 °C with shaking and subsequently harvested by centrifugation. The pellet resulting from a 1 liter culture was resuspended in 25 ml of buffer A (50 mM NaH_2_PO_4_/Na_2_HPO_4_, 300 mM NaCl, 10 mM imidazole and pH 8) plus protease inhibitor mixture (Complete™, Roche) and broken by French press. Following centrifugation at 12,000 × *g* for 40 min, the C-BgsA protein was predominantly present in the soluble fraction. The supernatant was loaded onto a His Trap HP column (5 ml, GE Healthcare) equilibrated with buffer A and eluted with a gradient of buffer B (50 mM NaH_2_PO_4_/Na_2_HPO_4_, 300 mM NaCl, 1 M imidazole and pH 8). Fractions containing C-BgsA were pooled and dialyzed against buffer A without imidazole. When required, C-BgsA was concentrated by using centrifugal filters Ultracel®-3K (Amicon-Millipore). Protein concentrations were determined using the Bio-Rad Protein Assay kit.

### Mutant library of C-BgsA by error-prone PCR

A mutant library of C-BgsA was constructed following a previously described method for random mutagenesis by error-prone PCR^46^. Briefly, the PCR was performed with an unbalanced ratio of nucleotides to minimize mutational bias in the amplified sequences using primers M91-F (GGTAGAGGGGTCGTTCTCGG) and M91-R (CAGGAATTCGCCCTTCTCC). The number of cycles and quantity of template DNA (pBBR5BgsA) was optimized to obtain a tractable number of average mutations per clone. The pool of mutant DNA fragments were purified and first cloned into pCR-XL-TOPO® (Invitrogen) to finally being subcloned *en mass* into pBBR5BcsA by exchanging the AsiSI/EcoRI fragment encoding the wild type C-BgsA segment (last 539 bp of the *bgsA* gene) for the random mutagenized C-BgsA PCR products. *E. coli* β2163 was used as donor strain to introduce the mutant library by conjugation into Sme *bgsA* Tn7pleD*Tc strain for complementation experiments.

### Deep sequencing of mutants by Illumina®

One thousand CR^+^ colonies was selected as a representative but still tractable number of mutants from the random mutagenized CR screening likely containing functional copies of BgsA. The mutants were picked into a flask with 500 ml of TY supplemented with Gm. A second flask with the same media was inoculated with Sme *bgsA* Tn7pleD*Tc containing the wild type pBBR5BcsA plasmid. Both flasks were grown with shaking until an early stationary phase. Plasmid DNA was extracted from 5 mL of each culture and sent to be sequenced by Illumina to the FISABIO foundation (Valencia, Spain). The coding sequence of C-BgsA was paired-end sequenced in both samples by using primers M91-F and M91-R2 (GCATCCCGCCCAACCTGC). After sequencing, the two ends were assembled and filtered to discard those that were incomplete ending with 2183 full-length sequences. To minimize the effect of artifacts raised by the intrinsic error of the sequencing technique, the values obtained in the control population (pBBR5BgsA) were subtracted from the values obtained for the corresponding positions in the mutant population (pBBR5BgsA*) and expressed in %.

### Calcofluor binding assays

To quantify MLG production, CF-derived fluorescence was measured in CF binding experiments. 500 μl of a starting culture in TY broth was washed twice with MM and diluted 1/100 into 10 ml flasks containing MM supplemented with CF (100 μM). Flasks were incubated for 48 h at 28 °C. Afterwards, cultures were centrifuged and supernatants with the unbound CF removed. The pellets were suspended in 2 ml distilled water and disposed in 24-well plates. Measures of four biological replicates from independent cultures for each strain were performed in a PTI fluorimeter (Photon Technology International). Statistical homoscedastic two-tailed student’s t-test was used to assess statistical differences across samples.

### Dot-blot experiments

Sonicated cell lysates (40 µg of total protein) or pure proteins (1.5 µg) were immobilized onto nitrocellulose membranes (Inmobilon®, Millipore) by dropping 4 µl of the different samples and let them air dry. Two sibling membranes were generated and blocked with TBS-T with 5% skimmed milk for 1 hour. One of the membranes was incubated for 1 hour with 1 µM of 2′-Fluo-AHC-c-di-GMP (Biolog, Cat. N°: F 009) in the blocking buffer at room temperature and developed after 3 × 5 min washes with TBS-T using a Phosphoimager FX (Bio-Rad) to analyze the c-di-GMP binding ability of the different samples. The second membrane was incubated for 1 hour at room temperature with an anti 6x-His epitope tag monoclonal antibody (Mouse/IgG; Thermo Scientific; Cat. N°: MA1-135) diluted 1:800 in the blocking buffer following manufacturers recommendations. After 5 × 5 min washes with TBS-T the membrane was incubated for 1 hour at room temperature with the secondary Anti-Mouse IgG, horseradish peroxidase linked antibody from sheep diluted 1:15000 in blocking buffer. The membrane was subsequently washed 5 × 5 min and developed with ECL^TM^ Prime Western Blotting detection Reagent (GE Healthcare) and photographed in a luminometer (Bio-Rad) to check the expression level of the tagged proteins in the different samples. The linearity of the cell lysate assay signal was confirmed by a parallel positive dot-blot control experiment with increasing amounts of the wild type cell lysates (0.06, 0.28, 1.4, 7.04 µg).

### SPR experiments

SPR experiments were performed using a Biacore 3000 instrument (Biacore, GE Healthcare) at 25 °C and a flow rate of 30 µl/min following the general principles described by Moll *et al*.^47^. Preparation of the 2′-AHC-c-di-GMP (Biolog, Cat. N°: F 151) sensor-surface was performed as described previously^48^. The reference cell was activated accordingly and subsequently deactivated without ligand immobilization. After testing different buffer conditions for C-BgsA, the assay buffer used in all SPR experiments was 20 mM MOPS pH 8.0, 300 mM NaCl, 0.005% P20 with 0.1 mg/ml of BSA. Direct binding assays with the immobilized 2′-AHC-c-di-GMP were performed by injecting increasing concentrations of C-BgsA (from 1.37 nM to 1 µM) to the sensor chip. After double referencing by subtracting the signal of the reference flow cell and the curve of a buffer injection, K_D_ value for C-BgsA binding to the 2′-AHC-c-di-GMP immobilized on the sensor chip surface was calculated using reference points at the end of the association phase and calculated with GraphPad Prism6 software. Solution competition assays were carried out by incubating indicated concentrations of C-BgsA with increasing concentrations of free c-di-GMP (Biolog, Cat. N°: F 0057; from 0 to 1 mM) for 2.5 hours and then injection over the 2′-AHC-c-di-GMP sensor chip surface. After each round of experiments, the different samples with 0 M c-di-GMP were re-injected to check the stability of the different proteins in the conditions tested. The association and dissociation phases were monitored for 2 minutes, respectively. The SPR signal was taken at the end of the association phase and plotted against the logarithm of each cyclic nucleotide concentration. EC_50_ values (half-maximal effective concentration) were calculated from sigmoidal dose response curves with GraphPad Prism 6 software. The EC_50_ value is the nucleotide concentration, which gives half-maximal competition of the SPR signal. Assuming a linear relationship between BgsA occupancy and response, the estimated EC_50_ is equal to the K_D_ (concentration where half-maximal BgsA is occupied). K_D_ and EC_50_ values of C-BgsA wild type come from two different set of purified proteins. EC_50_ values for wild type and mutants are means from 2 different technical replicas and are indicated as the means ± SD. SPR representative curves for the data are shown in the respective figures. After each protein injection, the sensor surfaces were regenerated by two injections of 0.05% SDS and the drifting baseline was stabilized by one injection of 1 M NaCl.

### Data Availability

The datasets generated during and/or analyzed during the current study are available from the corresponding author on reasonable request.

## Electronic supplementary material

Supplemental Information
