# Supplementary material for: A novel c-di-GMP binding domain in glycosyltransferase BgsA is responsible for the synthesis of a mixed-linkage β-glucan
Source: Sci Rep. 2017 Aug 21;7:8997. doi: 10.1038/s41598-017-09290-2 (PMC5567048; doi:10.1038/s41598-017-09290-2)
Supplement: Supplementary file 1 — Supplemental Information [file 41598_2017_9290_MOESM1_ESM.pdf]

**Supplementary Information**

# A novel c-di-GMP binding domain in glycosyltransferase BgsA is responsible for the synthesis of a mixed-linkage β-glucan

Daniel Pérez-Mendoza, Daniela Bertinetti, Robin Lorenz, María-Trinidad Gallegos, Friedrich W. Herberg and Juan Sanjuán

**Table S1. Bacterial strains and plasmids used in this work**

| Strains | Relevant characteristics | Reference |
|---|---|---|
| **Rhizobial strains** | | |
| *S. meliloti* 8530 | $ExpR^+$ derivative of Rm1021, $Sm^R$ | [1] |
| SMb20391::Nm | 8530 derivative with miniTn5 in *SMb20391*(*bgsA*); $Str^R$ $Nm^R$ | [2] |
| Sme *bgsA* Tn*7*pleD*Tc | SMb20391::Nm with a mini-Tn*7*pleD*Tc, $Tc^R$. Strain with high intracellular c-di-GMP levels due to the expression of the diguanylate cyclase *pleD** | This work |
| Sme *bgsA* Tn*7*Tc | 8530 mini-Tn*7*Tc, $Tc^R$. mini-Tn*7*pleD*Tc with a 1114 bp NcoI internal deletion of *pleD** | This work |
| ***E. coli*** **strains** | | |
| OmniMAX™ | [proAB lacIq lacZΔM15 Tn10(TetR ) Δ(ccdAB)] mcrA Δ(mrr hsdRMS-mcrBC) Φ 80(lacZ)ΔM15 Δ(lacZYA-argF)U169 endA1 recA1 supE44 thi-1 gyrA96 relA1 tonA panD | Invitrogen® |
| β2163 | *MG1655::ΔdapA::(erm-pir)RP4-2,Tc::Mu, $Km^r$, $Em^r$* | [3] |
| **Plasmids** | | |
| mini-Tn*7*pleD*Tc | mini-Tn*7*pleD* with 1.3 Kb KpnI fragment containing Tc marker $Ap^R$, $Tc^R$ | This work |
| mini-Tn*7*Tc | mini-Tn*7*pleD*Tc with a 1114 bp NcoI internal deletion, $Ap^R$, $Tc^R$ | This work |
| pUX-BF13 | Helper plasmid providing the Tn7 transposition functions in trans, $Ap^R$, $mob^+$, ori-R6K | [4] |
| pQE-80L | Medium-copy expression vector, IPTG-inducible T5 promoter, $Ap^r$ for N-terminus His6-tag fusions, $Ap^R$ | Qiagen® |
| pCR-XL-TOPO® | Cloning vector, $Km^R$ | Invitrogen® |
| pQE80L::C-BgsA | Expression vector encoding the last 139 amino acids of BgsA with a $His_6$-tag in the N-terminus | [2] |
| pBBR1MCS-5 | Cloning vector, $Gm^R$ | [5] |

| | | |
|---|---|---|
| pBBR5BgsA | pBBR1MCS-5 with a *bgsA* gene (Smb 20391) cloned ClaI/EcoRI, $Gm^R$ | This work |

## References


1 Pellock, B. J., Teplitski, M., Boinay, R. P., Bauer, W. D. & Walker, G. C. A LuxR homolog controls production of symbiotically active extracellular polysaccharide II by Sinorhizobium meliloti. *J.Bacteriol.* **184**, 5067-5076 (2002).

2 Pérez-Mendoza, D. *et al.* Novel mixed-linkage beta-glucan activated by c-di-GMP in *Sinorhizobium meliloti*. *Proc Natl Acad Sci U S A* **112**, E757-765, doi:10.1073/pnas.1421748112 (2015).

3 Demarre, G. *et al.* A new family of mobilizable suicide plasmids based on broad host range R388 plasmid (IncW) and RP4 plasmid (IncPalpha) conjugative machineries and their cognate *Escherichia coli* host strains. *Res.Microbiol.* **156**, 245-255 (2005).

4 Bao, Y., Lies, D. P., Fu, H. & Roberts, G. P. An improved Tn7-based system for the single-copy insertion of cloned genes into chromosomes of gram-negative bacteria. *Gene* **109**, 167-168 (1991).

5 Kovach, M. E. *et al.* Four new derivatives of the broad-host-range cloning vector pBBR1MCS, carrying different antibiotic-resistance cassettes. *Gene* **166**, 175-176 (1995).

## Sme C-BgsA

```
526-------536-------546-------556-------566-------576-------586-------596-------606-------616-------626-------636-------646-------656------
ASLICFEKPRRLLQAFKLDEPVDVDGVEGRLVSLSLDKAVVAVSTETRFKSTKVRLNIEGFAPLEADLKQVTQRRGDITRTGDKQRYYLHLHYDLRGVERDKMIIKLYTGRYSRDVPDIDKIAVSVNLLLRAFGRTRTA
---EE----HHHHHHH-------------EEEEEH----EEEE---------EEEEEE-------EHHHHH---------------EEEEEEEEE------EEEEEEE----------HHHHHHHHHHHHHHH------
9951126637888875257764104687357721016855875267777773578751788874002334206777772277612078888887035543206788862577777211018994494999982168999
```

## Rsp C-BcsA

```
RXXXR        (D/N)X(S/A)XXG
---581-------591-------601-------611-------621-------631-------641-------651-------661-------671-------681-------691-------701-------711-------721-------731-------741-------751-------761-------771-------781-----
QRRAAPRVQMEVPAEAQIPAFGNRSLTATVLDASTSGVRLLVRLPGVGDPHPALEAGGLIQFQPKFPDAPQLERMVRGRIRSARREGGTVMVGVIFEAGQPIAVRETVAYLIFGESAHWRTMREATMRPIGLLHGMARILWMAAASLPKTARDFMDEPARRRRRHEEPKEKQAHLLAFGTDFSTEPDWAGELLDPTAQVSARPNTVAWGSN
------------EEEEEE------EEEEEEEEEE---EEEEEE--------------EEEEEEEE-------EEEEEEEEEEEE-----EEEEEEE----HHHHHHHHHHHHH--HHHHHHHHH-------HHHHHHHHHHHHHHHHHHHHHHHHHH----------HHHHHHH-------------HHH---------------EEE---
0013156111550689971688717898999981065589982357777666556650689988137788771589988899982688268988862685689999999874066689999310127887528999999999999999999998621027666653103204200013147887763111111367777777762431489
```

PilZ domain

## Axy C-CeSA

```
RXXXR             (D/N)X(S/A)XXG
572-------582-------592-------602-------612-------622-------632-------642-------652-------662-------672-------682-------692-------702-------712-------722
QKRNSHRIPATIPVEVANADGSIIVTGVTEDLSMGGAAVKMSWPAKLSGPTPVYIRTVLDGEELILPARIIRAGNGRGIFIWTIDNLQQEFSVIRLVFGRADAWVDLGQLQGRPPAAQPHGHGSQRQGPVPFKWRYRPSQFPNQAFGWQCPV
-----EEEEEEEEEEEEE----EEEEEEEEEE---EEEEEE----------EEEEEEE----EEEEEEEEEEE---EEEEEE----HHHHHHHHHHHH----------------HHHHHHHHHHHHHHHHHHHHHH----------------
99971117331047998547862012111120577058885367777777448999854885589989999707865899840785476777766431357212677777776428999999999876899998525777777777777889
```

PilZ domain

**Figure S1. Secondary structure prediction of C-BgsA.** Prediction of the secondary structure of *Sinorhizobium meliloti* C-BgsA domain (526-664) compared with the last cytoplasmic segment of cellulose synthases from *Rhodobacter sphaeroides* (Rsp C-BcsA; 578-788) and *Komagataeibacter xylinum* (Axy C-CeSA; 572-723) containing the PilZ domains. Predictions were carried out by Jpred4 (Drozdetskiy A, Cole C, Procter J, Barton GJ. 2015.Nucleic Acids Research 43:W389-W394). The prediction classifies each amino acid residue as belonging to alpha helix ('H' in red), beta sheet ('E' in blue) or neither H or E ('-') secondary structures. The reliability of prediction accuracy is also indicated (score from 0 to 9, bigger is better). PilZ motifs RxxxR and (D/N)x(S/A)xxG relevant for c-di-GMP recognition are depicted above the sequences of cellulose synthases.

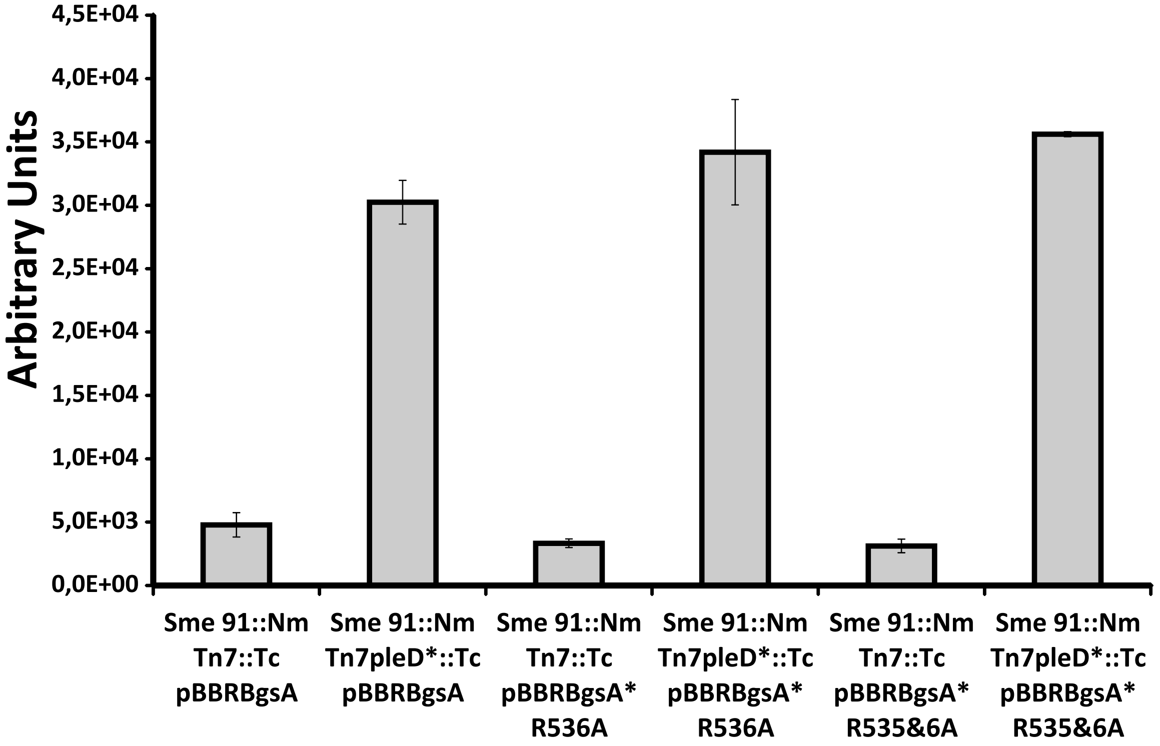


**Figure S2. MLG production.** Quantification of CF-derived fluorescence of *Sinorhizobium meliloti* 8530 bgsA under high (Tn7::Tc pleD*) and physiological (Tn7::Tc) c-di-GMP conditions, complemented with BgsA mutants in R536 or in both R535 and R536. Results are expressed in arbitrary units ± SE from four independent cultures.

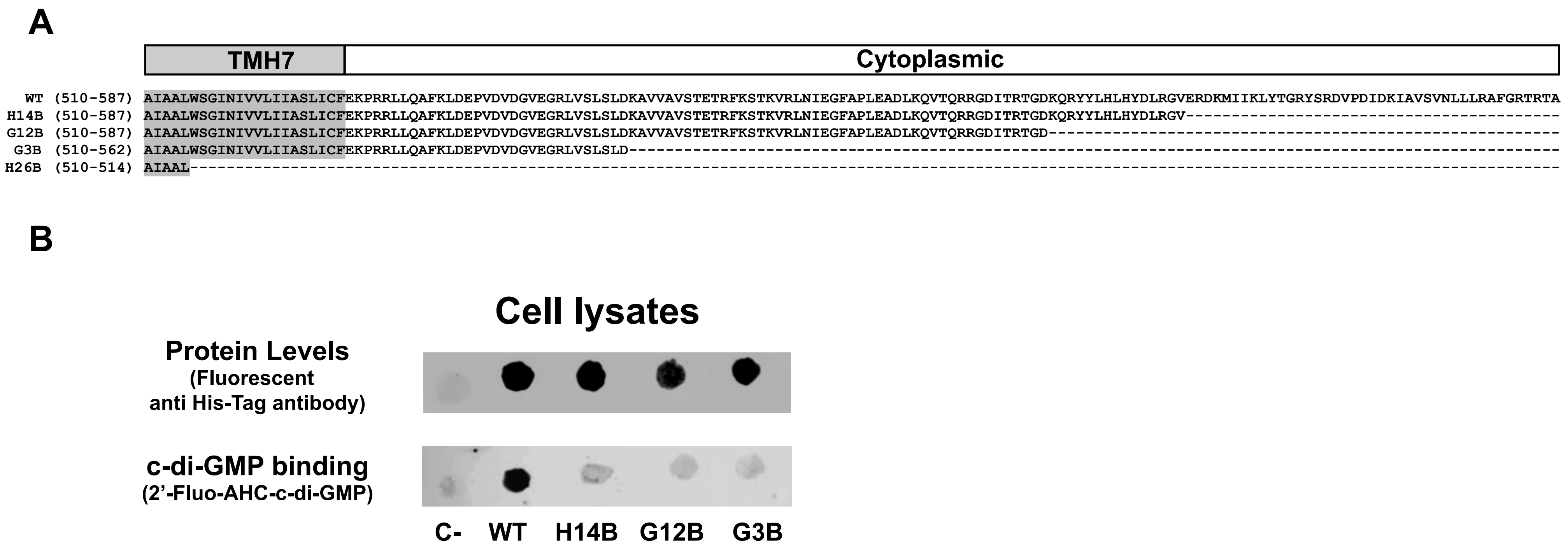


**Figure S3. c-di-GMP binding of E. coli lysates expressing different truncated versions of C-BgsA.** (A) Sequence of the different C-terminal truncated versions of BgsA. The last predicted transmembrane (TMH7) and C-terminal cytoplasmic segment of BgsA are indicated. (B) Dot-blot experiment with cellular extracts of the Omni Max *Escherichia coli* strain overexpressing the different truncated version and the wild type C-BgsA domains cloned in the pQE-80L vector. Two parallel dot-blots assays were performed by the incubation of 40 µg of the different cell lysates immobilized onto nitrocellulose membranes: (i) one incubated with an anti His-tag antibody for checking the abundance of the tagged protein in the different lysates; (ii) and the second incubated with 1µM of 2’-Fluo-AHC-c-di-GMP for 1 hour to assay for c-di-GMP binding. The linearity of the assay signal was confirmed in an additional dot-blot positive control with increasing amounts of the wild type lysate (See Methods).

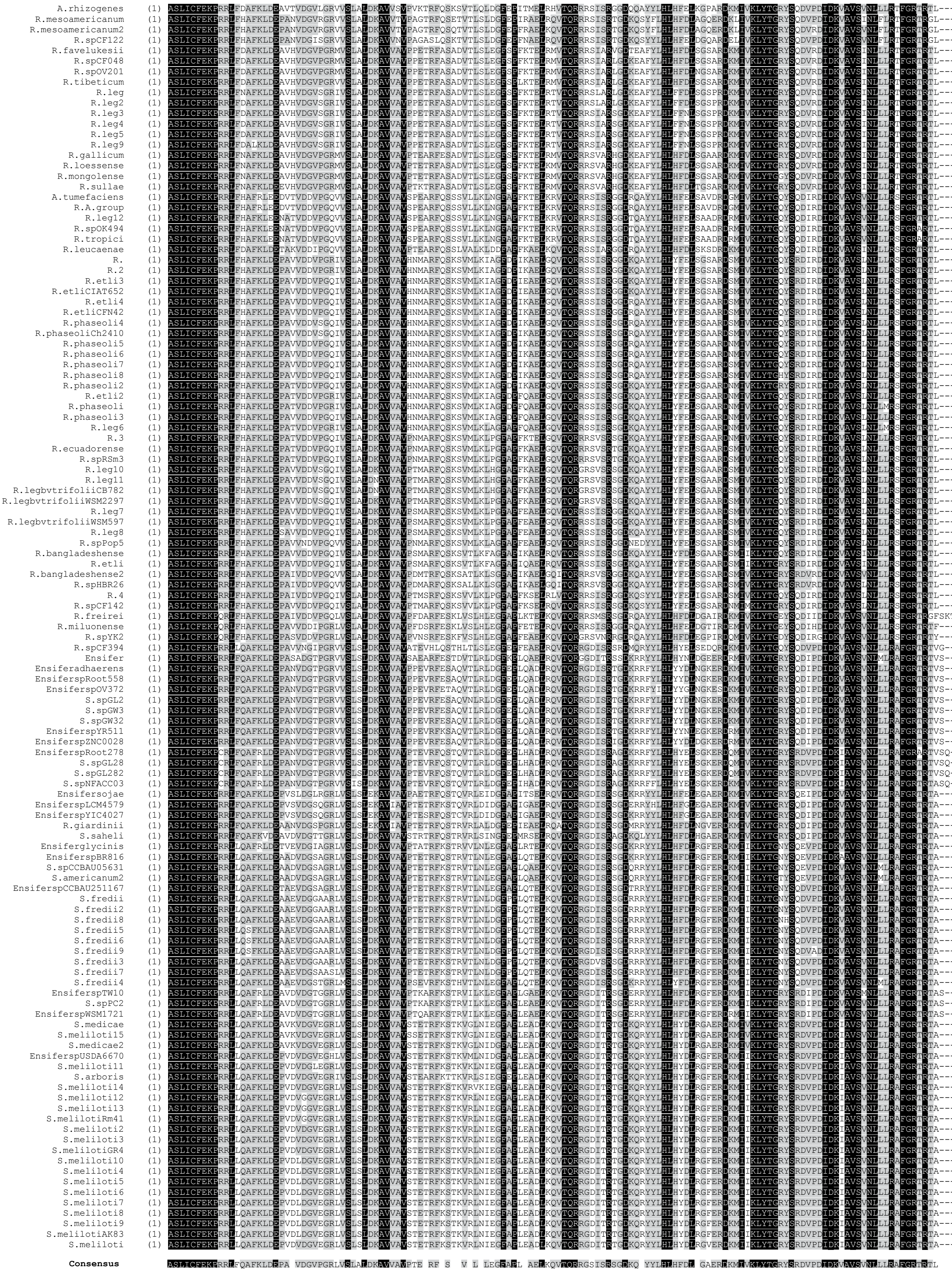

**Figure S4. Sequence alignment of the C-terminal segment of BgsA orthologues.** Predicted cytoplasmic C-BgsA domain sequences come from BgsA homologues available in NCBI database. Alignment was performed with Clustal W and the rhizobial species is indicated for each sequence. White on black back-ground = invariant residues; black on dark grey = strongly conserved and black on white = non-conserved.

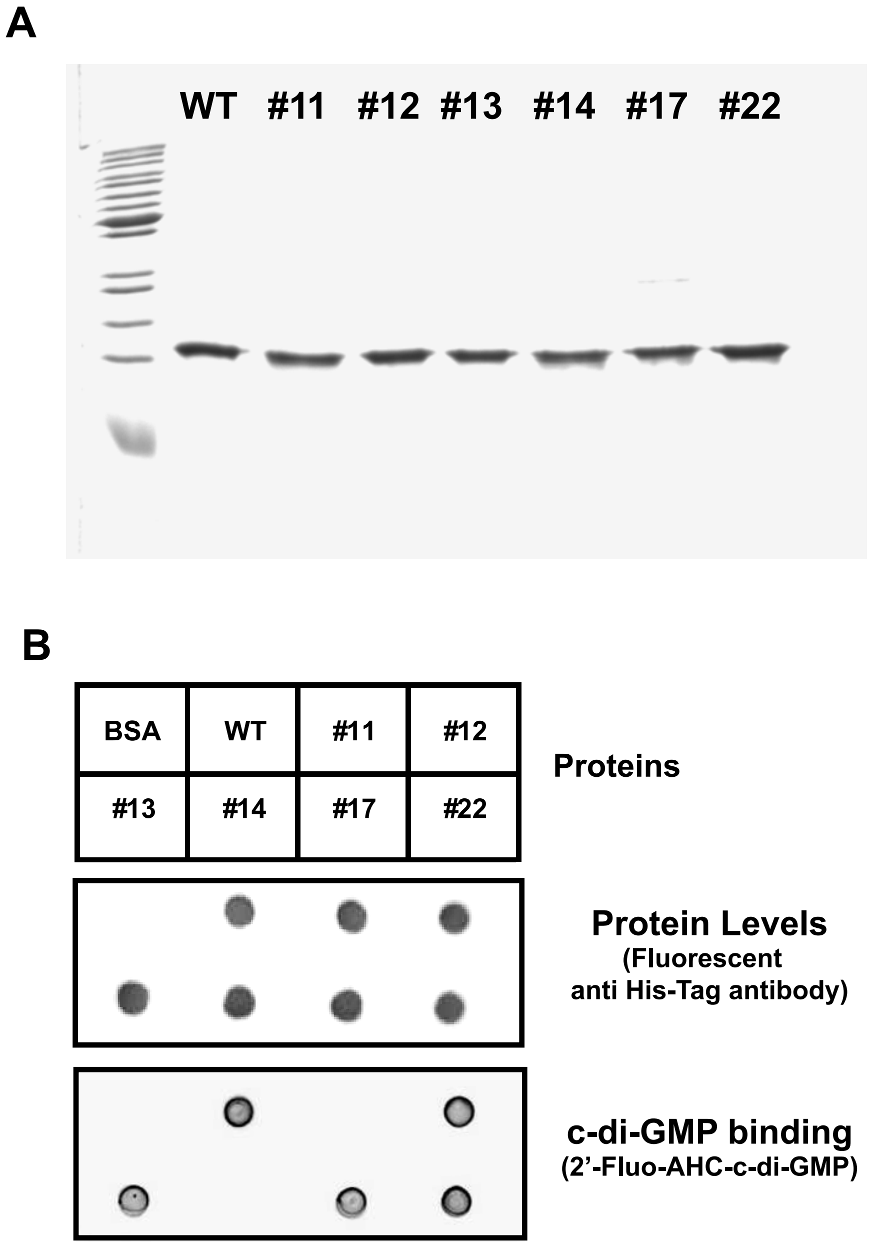


**Figure S5. Purification and c-di-GMP binding dot-blot assays of C-BgsA and selected mutants.** His tagged C-terminal domain of BgsA from the wild type and different selected mutants were overexpressed and purified by an affinity chromatography using a nickel column. (A) SDS-PAGE with a standardized amount of the wild type C-BgsA and the selected mutants stained with coomassie. (B) Dot-blot of 1,5 μg the purified proteins immobilized onto two different nitrocellulose membranes and incubated separately with (i) an anti His-tag antibody for checking the abundance of the tagged protein, and (ii) with 1μM of 2’-Fluo-AHC-c-di-GMP for 1 hour to assay for c-di-GMP binding.
